# Supplementary material for: The role of cryptic ancestral symmetry in histone folding mechanisms across Eukarya and Archaea
Source: PLoS Comput Biol. 2024 Jan 5;20(1):e1011721. doi: 10.1371/journal.pcbi.1011721 (PMC10796010; doi:10.1371/journal.pcbi.1011721)
Supplement: S1 Text — (PDF) [file pcbi.1011721.s001.pdf]

S1 Text for: The Role of Cryptic Ancestral Symmetry In Histone Folding Mechanisms Across Eukarya and Archaea

Haiqing Zhao[1,3¶*], Hao Wu[1], Alex Guseman[2], Dulith Abeykoon[2], Christina M. Camara[2], Yamini Dalal[3*], David Fushman[1,2*], Garegin A. Papoian[1,2*]

1 Biophysics Program, Institute for Physical Science and Technology, University of Maryland, College Park, Maryland, United States
2 Department of Chemistry and Biochemistry, University of Maryland, College Park, Maryland, United States
3 Laboratory of Receptor Biology and Gene Expression, National Cancer Institute, National Institutes of Health, Bethesda, Maryland, United States

¶Current address: Department of Systems Biology, Columbia University, New York, New York, United States
* hz2592@columbia.edu (HZ); fushman@umd.edu (DF); dalaly@mail.nih.gov (YD); gpapoian@umd.edu (GAP)

Contents

1 Method and Analysis Details **2**
1.1 AWSEM-MD Simulations 2
1.2 All-atom MD Simulations 2
1.3 Order Parameter Q 3
1.4 Polymer Scaling Analysis 3

2 Additional Discussion for Histone Folding/Binding Mechanism and Complex Structure Prediction **4**

1 Method and Analysis Details

1.1 AWSEM-MD Simulations

In this work, we used the AWSEM [1] model to simulate all the histone and histone fold protein (HFP) systems. The parameters in AWSEM were tuned such that the simulated melting temperature (*i.e.* the temperature at which both the folded and unfolded states are equally populated at equilibrium) of histone dimers is around 350 K, as observed in experiments. In addition, we employed an AWSEM-featured bioinformatic term called "fragment memory", using available protein segments as local structural bias. In histone/HFP monomer annealing simulations, the biasing segments were selected from proteins in the PDB which share similar local amino acid sequences to the histone monomers, equivalent to the "homologue allowed" structural library used in Davtyan *et al.* [1]. In the dimer simulations, the local memory fragments were generated from the histone monomer of histone complex or nucleosome X-ray crystal structures (PDB IDs in the next paragraph). The length of a local fragment is typically from 3 to 9 residues. They only provide local structural information, with no tertiary contacts within each monomer and no contact information between any two monomers (as used in previous protein binding studies with AWSEM [2]).

We ran AWSEM simulations using the open-source molecular dynamics software, LAMMPS [3] (version 9Oct12), with non-periodic shrink-wrapped boundary condition and the Nose-Hoover thermostat. The simulation time step was set as 5 femtoseconds. The initial conformations of the unfolded state were prepared at 1000K. All annealing simulations started from the completely unfolded state, and then were slowly cooled down from 600 K to 200 K. The simulation time of a production run is 1×10^7 steps. Ten independent runs with different initial states and velocities were performed for each system. The native conformations were taken from the corresponding X-ray crystal structures (PDB: 1AOI [4] for histone H2A/H2B and H3/H4; PDB: 3R45 [5] for CENP-A/H4; PDB: 1B67 and 1A7W [6] for archaeal histone HmfA and HmfB; PDB: 1TAF [7] for $\mathrm{dTAF_{II}}$; PDB: 1N1J [8] for NF-Y). In current study, we focus on understanding the histone fold, so histone tails and N- and C-terminal helices are typically excluded in our simulations. The sequences of proteins used here can be found in the following multiple sequence alignment figure (Figure **??**).

To calculate the free energy profile, we used umbrella sampling. The Q of the simulated molecules is chosen as the reaction coordinate. We set up 19 umbrella windows along Q ranging from 0 to 1. A harmonic potential around each Q_0 was added to the total Hamiltonian as in the equation $V(Q) = \frac{\kappa}{2}(Q - Q_0)^2$. The spring constant κ that we used here is 1000 kcal/mol/Å^2. In each window, the initial conformation is prepared by annealing under the Q_o potential biase procedures from 450 K to 250 K. The final umbrella sampling is at 300 K. Weighted histogram analysis method (WHAM) was used to remove the potential biase and calculate the free energy profiles.

1.2 All-atom MD Simulations

The all-atom simulations were performed in the high-performance MD engine OpenMM 7.6.0 [9], with the input files prepared by CHARMM-GUI [10]. The atomic force fields used in this work include Amber ff14SB force field for protein, the TIP3P water model for solvent, and the Joung/Cheatham ion parameters [11] for TIP3P water. Particle-Mesh-Ewald (PME) electrostatics and switched Leonard-Jones interactions with a cutoff distance of 12 Å were used in all the simulations. The protein systems to be simulated were solvated in 150 mM KCl solution, with periodical boundary conditions of a minimum distance of 12 Å. The timestep was set up at 2 fs. The energy minimization takes 50,000 timesteps, followed by equilibration under the NVT ensemble at 300 K for

500,000 timesteps. Hereafter the production runs were performed for 400,000,000 steps (800 ns) under the NPT ensemble, using Langevin thermostat and MonteCarlo barostat. Along each trajectory, atomic coordinates were saved every 25,000 steps.

For each simulated system, we run the abovementioned process for two independent replicas using different random seeds for their initial velocity states. The simulated systems are: 1). H2AH2B from the X-ray crystal structure (PDB: 1AOI) (trancated-sequence, chain A: P26-D90, chain B: Y34-L98); 2). AWSEM-predicted H2AH2B inverted non-native structure (trancated-sequence, chain A: P26-D90, chain B: Y34-L98); 3). AWSEM-predicted H2AH2A homodimer structure (trancated-sequence, P26-D90, P26-D90); 4). AlphaFold2-predicted H2AH2A homodimer structure (trancated-sequence, P26-D90, P26-D90); 5). AlphaFold2-predicted H2AH2A full-sequence homodimer structure (G4-K11, G4-K11); 6). Full-sequence H2AH2B from X-ray crystal structure (PDB: 1AOI) (G4-K118, K24-K122). In total, we run 9600 ns all-atom explicit-solvent simulations for different histone dimer structures.

Before running all-atom simulations, conformations from AWSEM predictions were refined using FoldX (v5.0) [12] to repair and optimize the side-chain atomic structures, and then using Chiron [13] to further remove steric clashes. Conformations from AlphaFold2 predictions were relaxed with amber force fields as implemented in the AlphaFold2 protocal.

1.3 Order Parameter Q

To quantitatively describe the similarity between simulated and native structures, we used the order parameter Q defined as in Davtyan *et al.* [1]:

$$Q = \frac{1}{N_{pairs}} \sum_{i<j-2} \exp[-\frac{(r_{ij} - r_{ij}^N)^2}{2\sigma_{ij}}] \tag{1}$$

where N_{pairs} is the number of pairs in the summation, r_{ij} is the instantaneous distance between C_α atoms of residues i and j, r_{ij}^N is the same distance in the native structure, and $\sigma_{ij} = (1 + |i - j|)^{0.15}$ represents the resolution of distance difference.

The range of Q is from 0 to 1. A higher Q value means that the simulated conformation is more similar to the native structure. Note that the group of atoms included for computing Q can be customized. In the main text, we computed $Q_{monomer}$ using the C_α atoms only within a monomer, while Q_{dimer} was calculated using the entire dimer.

1.4 Polymer Scaling Analysis

Our computer simulations and NMR and CD experiments suggest that histone dimers should be considered as an independent folding unit. From a perspective of polymer physics, the radius of gyration for a polymer chain (R_g) approximately follows the scaling relation: $R_g \sim \alpha N^\nu$, where N is the number of bond segments (*i.e.* the degree of polymerization) of the chain, α is the linear slope, and ν is the scaling exponent. Dima and Thirumalai [14] estimated this scaling relation for proteins after analyzing a large dataset of monomeric protein structures. Based on their obtained values of $\alpha \simeq 3$ and $\mu \simeq 1/3$, we fitted the R_g and N of the X-ray crystal structures of histone monomers and dimers (Fig 1). We found that all the histone monomers have higher R_g than expected for a globular protein with the same residue length N. Histone dimers, on the other hand, follow very well the R_g trend of single domain proteins, again supporting the idea that histone dimers represent a single folding unit.

2 Additional Discussion for Histone Folding/Binding Mechanism and Complex Structure Prediction

From the protein folding and binding theory perspective, a previous study by Levy *et al.* [15] suggested that as in protein folding, the native topology of proteins is the major factor that determines their folding upon binding mechanism. In the scenario of histones, the "hand-shake" geometry of histone-fold structure implies that histone may adopt an "induced-fit" mechanism model of protein-protein association [16]. Thus, it may not be surprising to realize structures like histone fold to have an induced-fit, or a coupled folding and binding mechanism. On the other hand, the unique sequence symmetry of histones with conserved hydrophobic residues lead the two participating monomers to fit with the induced formation but in two different ways, wherein the reserved hydrophobic residues play a dominant role. Interestingly, a previous experimental study found that H2A and H2B first rapidly recognize each other to form two intermediate bound states via weak hydrophobic interactions, and then rearrange to fold as the native structure [17]. This finding supports our hypothesis that the symmetrical hydrophobic interactions is essential for the induced-fit folding and binding process of histones. Another work by Zhou and coworkers showed that the foldability of a protein with native-reversed sequence depends on the protein size and location of its native hydrophobic core [18]. Here, in the histone binding example, we see that the conserved hydrophobic positions supports the N-C-terminal flipped binding conformations between two histones.

Protein structure prediction has been a long challenging problem since 1970s. Recently, breakthrough has been made through deep-learning based algorithms [19, 20] and it is viewed as a stunning advance on solving the protein-folding problem. In this work, to predict homodimer structure of histones we applied the cutting-edge deep-learning based algorithm AlphaFold in ColabFold [21]. ColabFold is an online platform for protein folding and homo- and heteromer complex folding. Two models from AlphaFold2 were adpated: one is the original AlphaFold2 model [20] which was trained for monomeric protein folding; the other being the AlphaFold-Multimer [22] which is an AlphaFold2 model but trained specifically for multimeric inputs of known stoichiometry.

Using ColabFold-adapted AF2 monomer model, we were able to predict complex for the H2A/H2A and got the native-like histone fold for H2A/H2A, while using AlphaFold-multimer, we obtained the inverted crossing way of histone-fold structure as what we found in AWSEM simulations (Fig S13). In total, the predictions of AF2 show outstanding consistency with that of AWSEM, which is mostly based on protein folding funnel energy landscape theory and coarse-grained MD simulations. The fact of different predictions out of two versions of AlphaFold approaches highlights the importance of training process in such methods. It is also possible that the AF-multimer is overtrained on available protein structures and have missed other possible states. One recent study indicates that modifying the multiple sequence alignment depth with stochastic subsampling may help generate alternative conformations [23], and another work points that optimising the multiple sequence alignment improves the precision of protein-protein interaction predictions [24].

As it is known, AlphaFold2 is an end-to-end protein structure prediction algorithm based on protein sequences and native protein structures, where the folding dynamics is completely absent. Together with our MD simulations results at both coarse-grained and all-atom resolutions, this work suggests that histone tails can increase the formation energy barrier of histone homo-dimerization likely by perturbing the structural fitting of histone-fold hand-shake core. Yet, once the homodimer has overcome the energy barrier, histone-fold structure may be formed and stay as stable.

References

1. Davtyan A, Schafer NP, Zheng W, Clementi C, Wolynes PG, Papoian GA. AWSEM-MD: protein structure prediction using coarse-grained physical potentials and bioinformatically based local structure biasing. The Journal of Physical Chemistry B. 2012;116(29):8494–8503.

2. Zheng W, Schafer NP, Davtyan A, Papoian GA, Wolynes PG. Predictive energy landscapes for protein–protein association. Proceedings of the National Academy of Sciences. 2012;109(47):19244–19249.

3. Plimpton S. Fast parallel algorithms for short-range molecular dynamics. Journal of Computational Physics. 1995;117(1):1–19.

4. Luger K, Mäder AW, Richmond RK, Sargent DF, Richmond TJ. Crystal structure of the nucleosome core particle at 2.8 Å resolution. Nature. 1997;389(6648):251.

5. Hu H, Liu Y, Wang M, Fang J, Huang H, Yang N, et al. Structure of a CENP-A–histone H4 heterodimer in complex with chaperone HJURP. Genes & development. 2011;25(9):901–906.

6. Decanniere K, Babu AM, Sandman K, Reeve JN, Heinemann U. Crystal structures of recombinant histones HMfA and HMfB from the hyperthermophilic archaeon Methanothermus fervidus. Journal of molecular biology. 2000;303(1):35–47.

7. Xie X, Kokubo T, Cohen SL, Mirza UA, Hoffmann A, Chait BT, et al. Structural similarity between TAFs and the heterotetrameric core of the histone octamer. Nature. 1996;380(6572):316–322.

8. Romier C, Cocchiarella F, Mantovani R, Moras D. The NF-YB/NF-YC structure gives insight into DNA binding and transcription regulation by CCAAT factor NF-Y. Journal of Biological Chemistry. 2003;278(2):1336–1345.

9. Eastman P, Swails J, Chodera JD, McGibbon RT, Zhao Y, Beauchamp KA, et al. OpenMM 7: Rapid development of high performance algorithms for molecular dynamics. PLoS computational biology. 2017;13(7):e1005659.

10. Jo S, Kim T, Iyer VG, Im W. CHARMM-GUI: a web-based graphical user interface for CHARMM. Journal of computational chemistry. 2008;29(11):1859–1865.

11. Joung IS, Cheatham III TE. Determination of alkali and halide monovalent ion parameters for use in explicitly solvated biomolecular simulations. The journal of physical chemistry B. 2008;112(30):9020–9041.

12. Delgado J, Radusky LG, Cianferoni D, Serrano L. FoldX 5.0: working with RNA, small molecules and a new graphical interface. Bioinformatics. 2019;35(20):4168–4169.

13. Ramachandran S, Kota P, Ding F, Dokholyan NV. Automated minimization of steric clashes in protein structures. Proteins: Structure, Function, and Bioinformatics. 2011;79(1):261–270.

14. Dima RI, Thirumalai D. Asymmetry in the shapes of folded and denatured states of proteins. The Journal of Physical Chemistry B. 2004;108(21):6564–6570.

15. Levy Y, Wolynes PG, Onuchic JN. Protein topology determines binding mechanism. Proceedings of the National Academy of Sciences. 2004;101(2):511–516.

16. Koshland Jr DE. The key–lock theory and the induced fit theory. Angewandte Chemie International Edition in English. 1995;33(23-24):2375–2378.

17. Guyett PJ, Gloss LM. The H2A–H2B Dimeric Kinetic Intermediate Is Stabilized by Widespread Hydrophobic Burial with Few Fully Native Interactions. Journal of molecular biology. 2012;415(3):600–614.

18. Zhang Y, Weber JK, Zhou R. Folding and stabilization of native-sequence-reversed proteins. Scientific reports. 2016;6(1):1–7.

19. Wang S, Sun S, Li Z, Zhang R, Xu J. Accurate de novo prediction of protein contact map by ultra-deep learning model. PLoS computational biology. 2017;13(1):e1005324.

20. Jumper J, Evans R, Pritzel A, Green T, Figurnov M, Ronneberger O, et al. Highly accurate protein structure prediction with AlphaFold. Nature. 2021;596(7873):583–589.

21. Mirdita M, Schütze K, Moriwaki Y, Heo L, Ovchinnikov S, Steinegger M. ColabFold-Making protein folding accessible to all. 2021;.

22. Evans R, O'Neill M, Pritzel A, Antropova N, Senior AW, Green T, et al. Protein complex prediction with AlphaFold-Multimer. BioRxiv. 2021;.

23. del Alamo D, Sala D, Mchaourab H, Meiler J. Sampling the conformational landscapes of transporters and receptors with AlphaFold2. bioRxiv. 2021;.

24. Bryant P, Pozzati G, Elofsson A. Improved prediction of protein-protein interactions using AlphaFold2 and extended multiple-sequence alignments. BioRxiv. 2021;.
