# Supplementary figures and images for: The role of cryptic ancestral symmetry in histone folding mechanisms across Eukarya and Archaea
Source: PLoS Comput Biol. 2024 Jan 5;20(1):e1011721. doi: 10.1371/journal.pcbi.1011721 (PMC10796010; doi:10.1371/journal.pcbi.1011721)

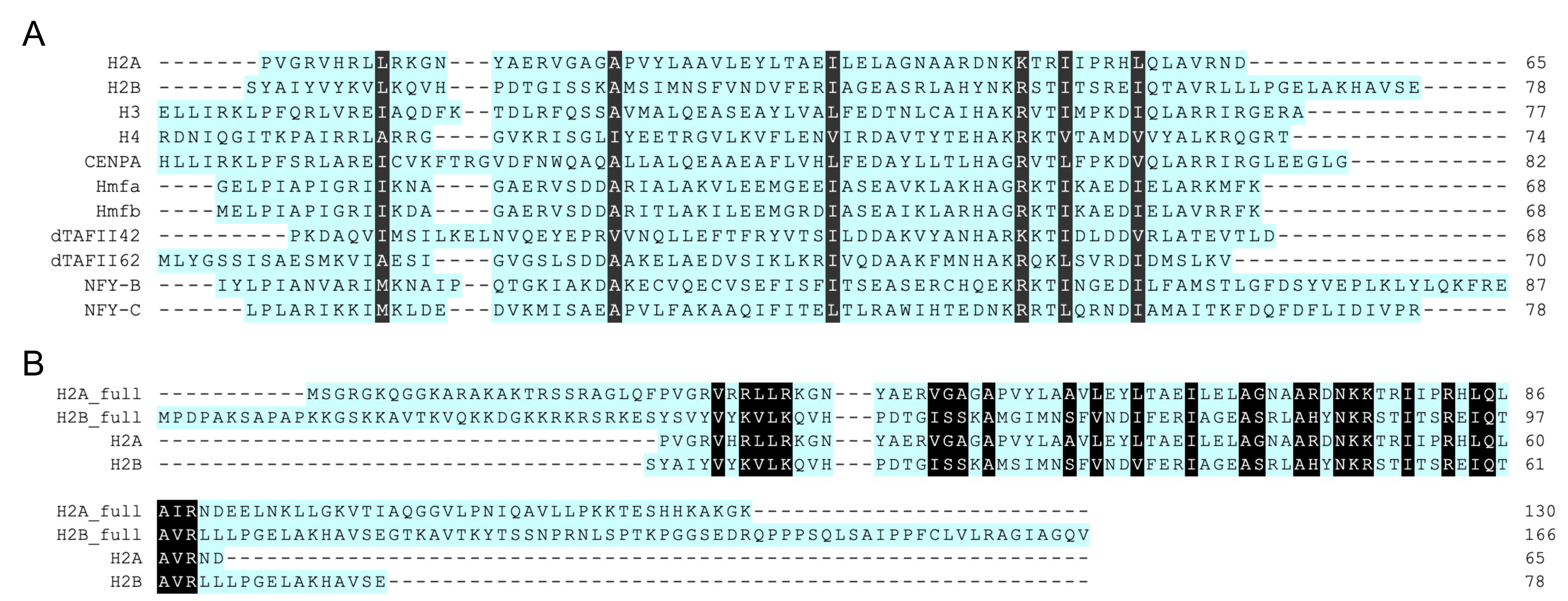

Supplement: S1 Fig — (A) MSA of the histone and histone-like proteins in this study show that the conserved residues (highlighted in black shade) are mostly hydrophobic and located near the dimer interfaces. (B) Full sequences of H2A and H2B aligned with histone-fold core of H2A and H2B, highlighting the extra length of histone tails on both N-terminal and C-terminal ends. (TIF) [file pcbi.1011721.s003.tif]

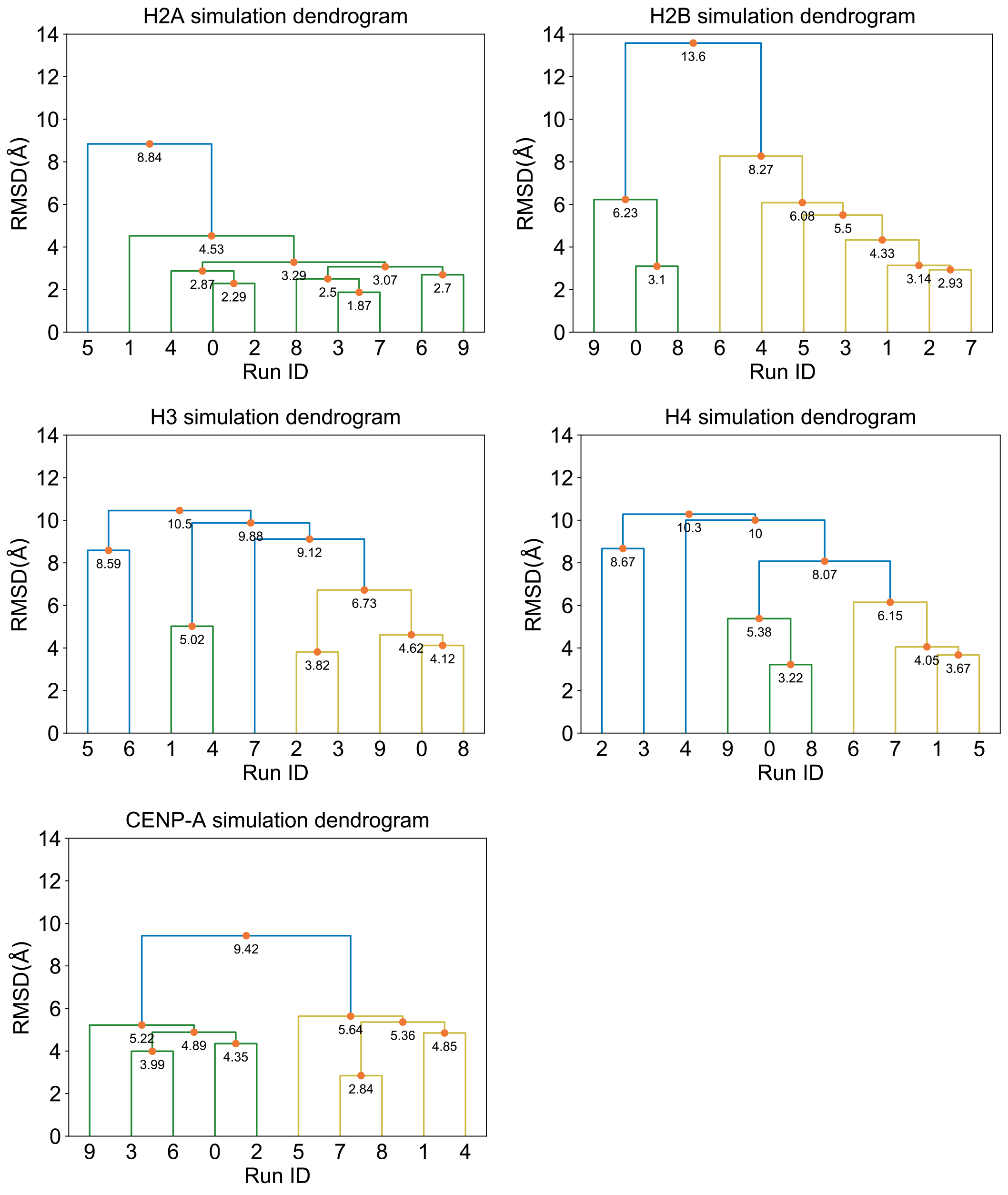

Supplement: S2 Fig — (A) Complementary to Fig 1B in the main context, here are simulation results for monomers of archaeal histone and two types of transcription factor dTAF and NF-Y. (B) Structure alignments based on the longest α2 helix (orange) for monomer simulation of H2A, H3, HMfA and NF-YC. Only the final conformations in corresponding annealing runs are included. (TIF) [file pcbi.1011721.s004.tif]

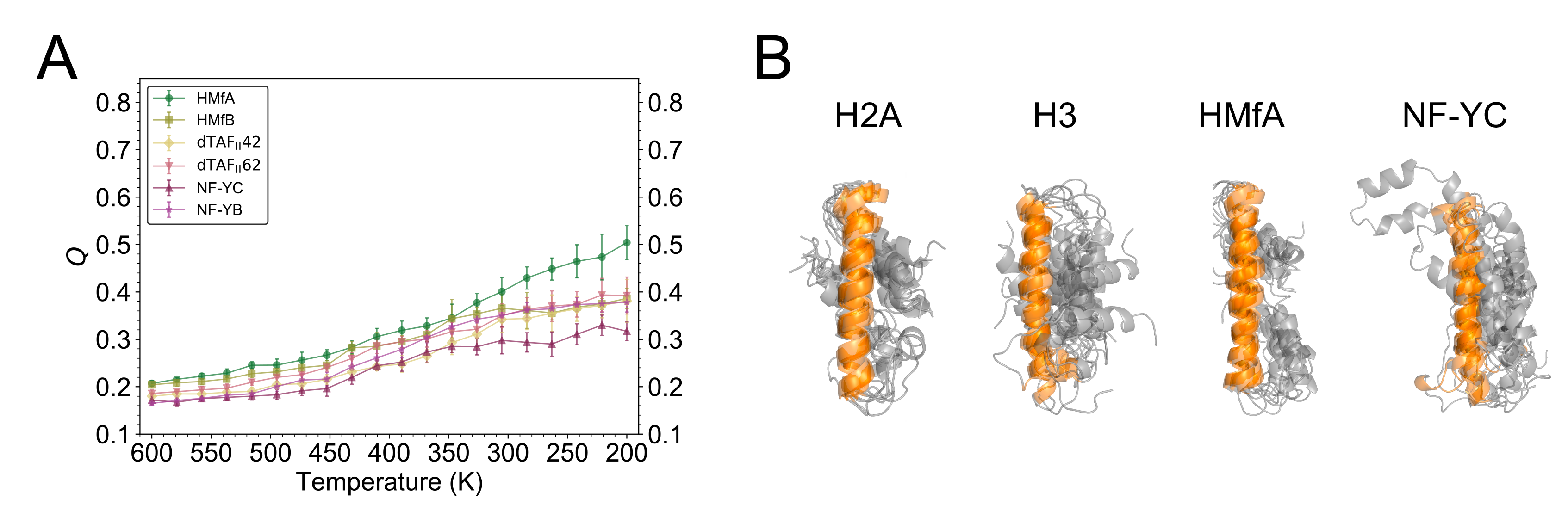

Supplement: S3 Fig — Shown here are the clustering analysis results based on the pairwise RMSD among ten independent annealing runs for each histone monomer. No consensus structure was found. (TIF) [file pcbi.1011721.s005.tif]

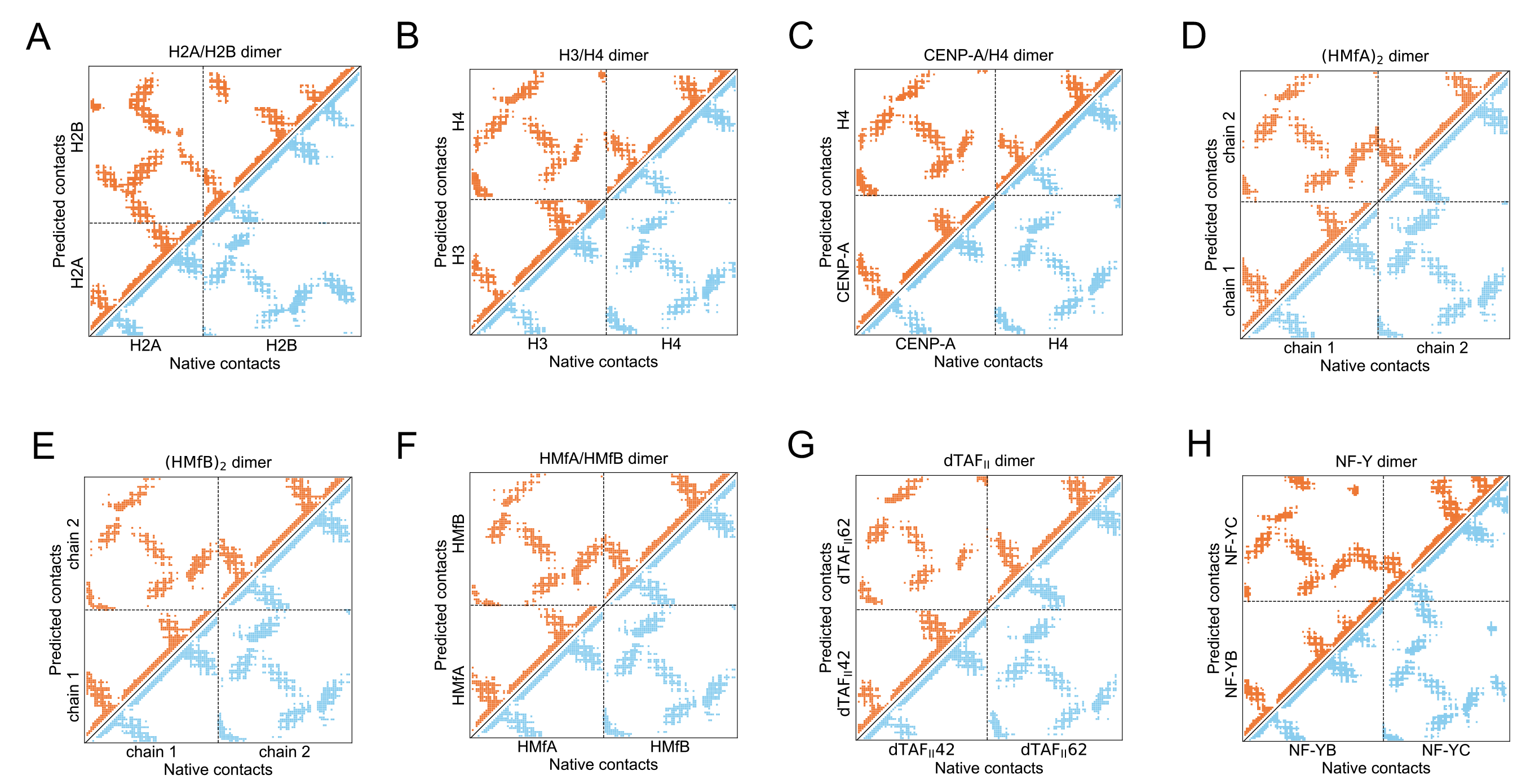

Supplement: S4 Fig — Contact maps are plotted for all the histone-like protein dimers in this study: (A) H2A/H2B; (B) H3/H4; (C) CENP-A/H4; (D) (HMfA)2; (E) (HMfB)2; (F) HMfA/HMfB; (G) dTAFII42/dTAFII62; (H) NF-YB/NF-YC dimer. Native and predicted contacts are represented in blue and orange, respectively. Predicted contacts for each dimer are computed using the structure of a simulation snapshot with the highest Q value from ten annealing runs. (TIF) [file pcbi.1011721.s006.tif]

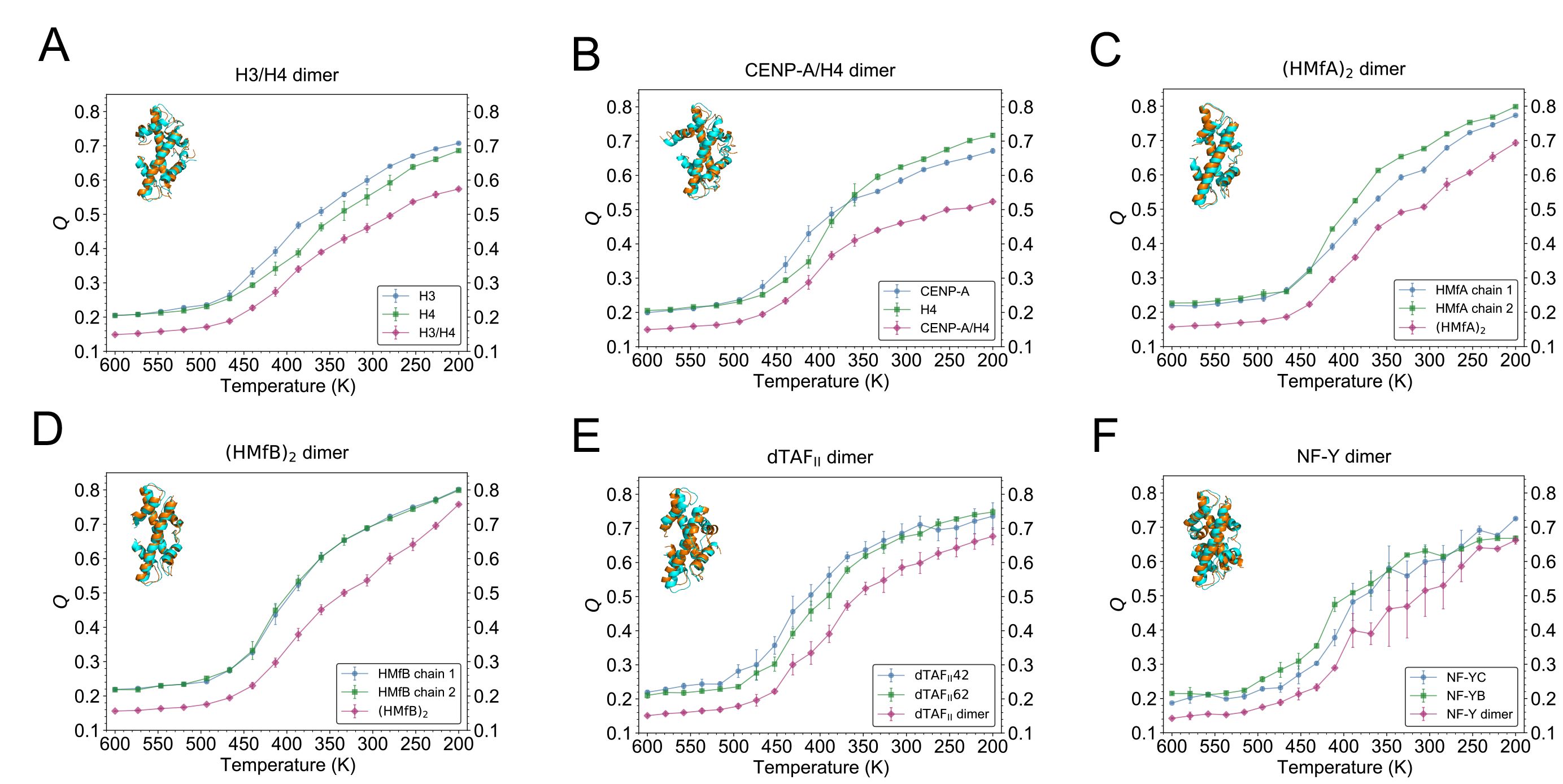

Supplement: S5 Fig — Q values for the folded monomer and dimer are shown as functions of the annealing temperature in AWSEM-MD simulations of H3/H4 (A), CENP-A/H4 (B), (HMfA)2 (C), (HMfB)2 (D), and histone-like dimers dTAFII42/dTAFII62 (E), NF-YB/NF-YC (F). Markers and error bars represent the mean values and standard deviations of Q from ten independent simulation runs. (TIF) [file pcbi.1011721.s007.tif]

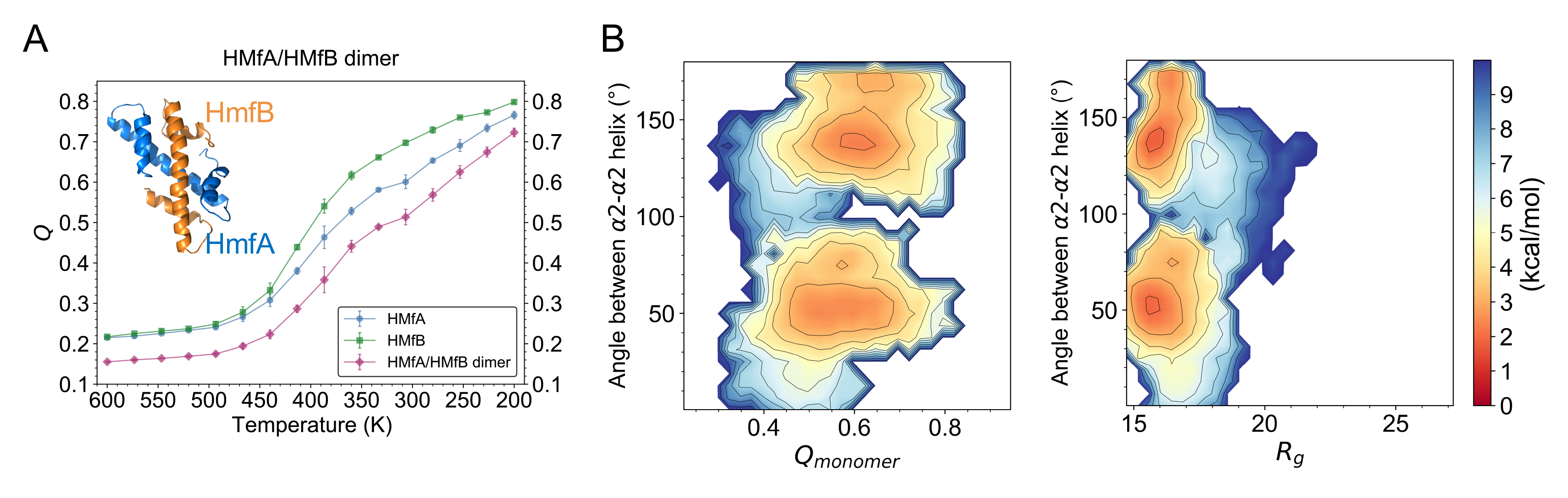

Supplement: S6 Fig — Previous biochemical studies demonstrate the formation of archaeal heterodimers HMfA/HMfB in vitro, which, however, was not structurally characterized. Based on our finding of eukaryotic histones (Fig 2), we hypothesize that archaeal histone heterodimers adopt a structure and binding-folding mechanism analogous to other eukaryotic histone dimers. We took HMfA and HMfB monomer structures from their homodimer complexes (PDB: 1B67 and 1A7W), and predicted their heterodimer structure using simulated annealing. We further applied umbrella sampling and calculated its free energy profiles. (A) Q value of the archaeal histone HMfA (blue) and HMfB (green), and its dimer (magenta) during the simulated annealing and the final prediction of archaeal heterodimer HMfA/HMfB structure are shown. (B) Free energy profile calculated at 300 K are projected on Qmonomer and the α2—α2 angle (left), and radius of gyration Rg and the α2—α2 angle (right). (TIF) [file pcbi.1011721.s008.tif]

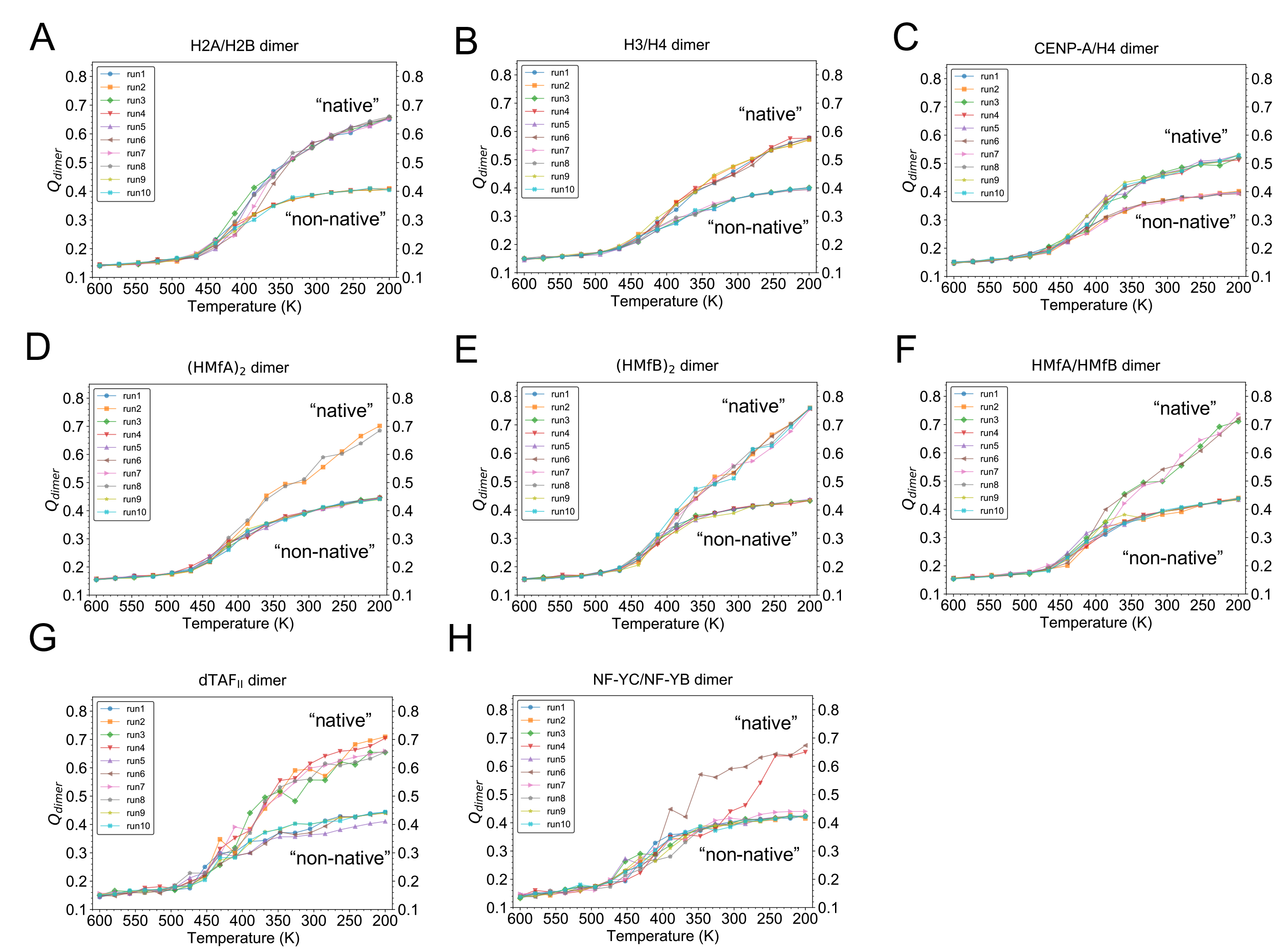

Supplement: S7 Fig — 15N{1H} SOFAST-HMQC spectra of 15N-labeled H2B alone (A) and in the presence of unlabeled H2A at an equimolar ratio (B). (C) Heteronuclear steady-state 15N{1H} NOE spectra recorded with amide proton presaturation for 15N-labeled H2B in the presence of unlabeled H2A at an equimolar ratio. In these spectra contours with positive intensities are colored black while negative intensities are blue. (TIF) [file pcbi.1011721.s009.tif]

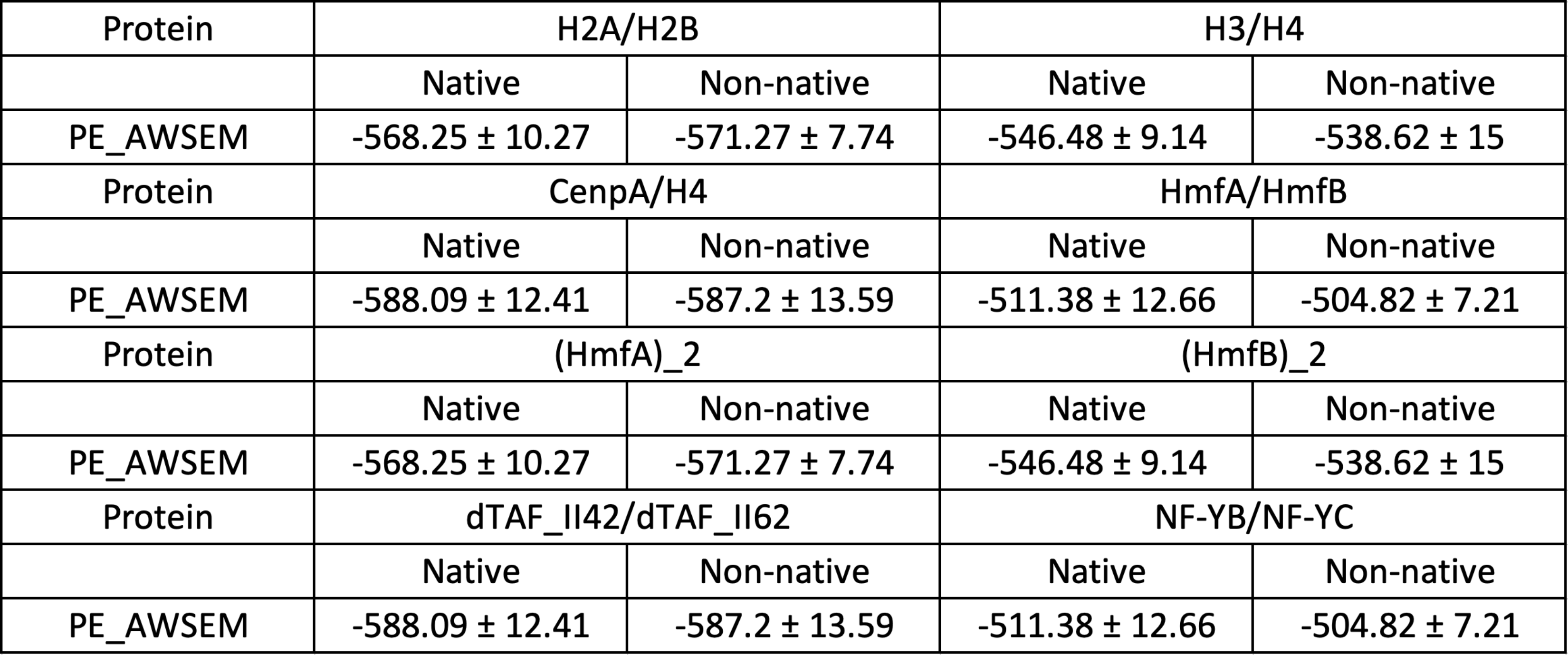

Supplement: S8 Fig — Qdimer values are shown as functions of the annealing temperature for AWSEM-MD simulations of H2A/H2B (A), H3/H4 (B), CENP-A/H4 (C), (HMfA)2 (D), (HMfB)2 (E), HMfA/HMfB (F) and dimers dTAFII42/dTAFII62 (G), NF-YB/NF-YC (H). Ten individual simulation runs are represented in different colors and marker types. We categorize all the runs with a final Q>0.5 as “native” and Q<0.5 as “non-native”. (TIF) [file pcbi.1011721.s010.tif]

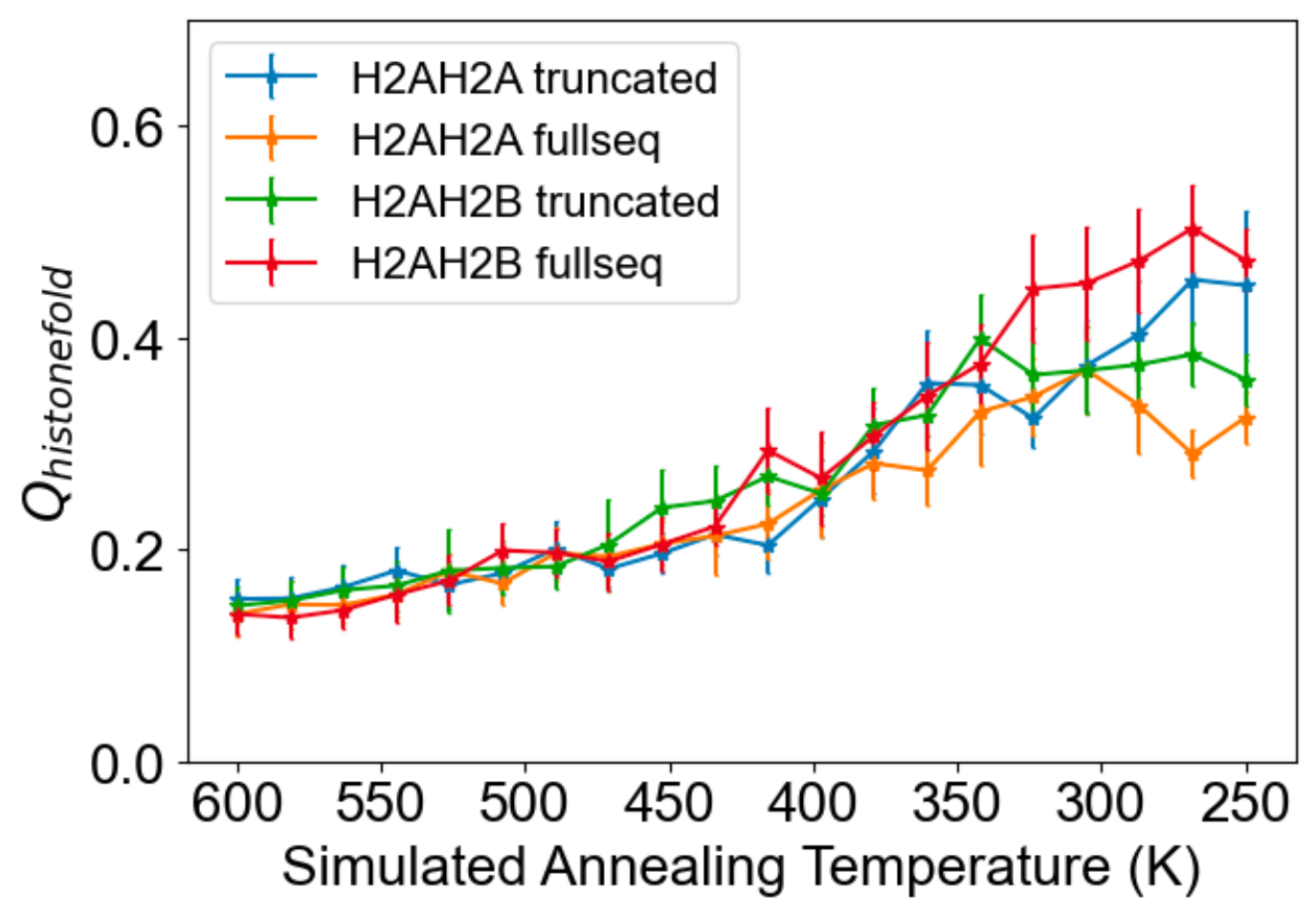

Supplement: S9 Fig — The potential energy in AWSEM-MD includes the follow terms: Vbackbone, Vcontact, Vburial, VHB, VAM and VDSB with details described in Davtyan et al. [23]. Numbers in this table are in the unit of kcal/mol. (TIF) [file pcbi.1011721.s011.tif]

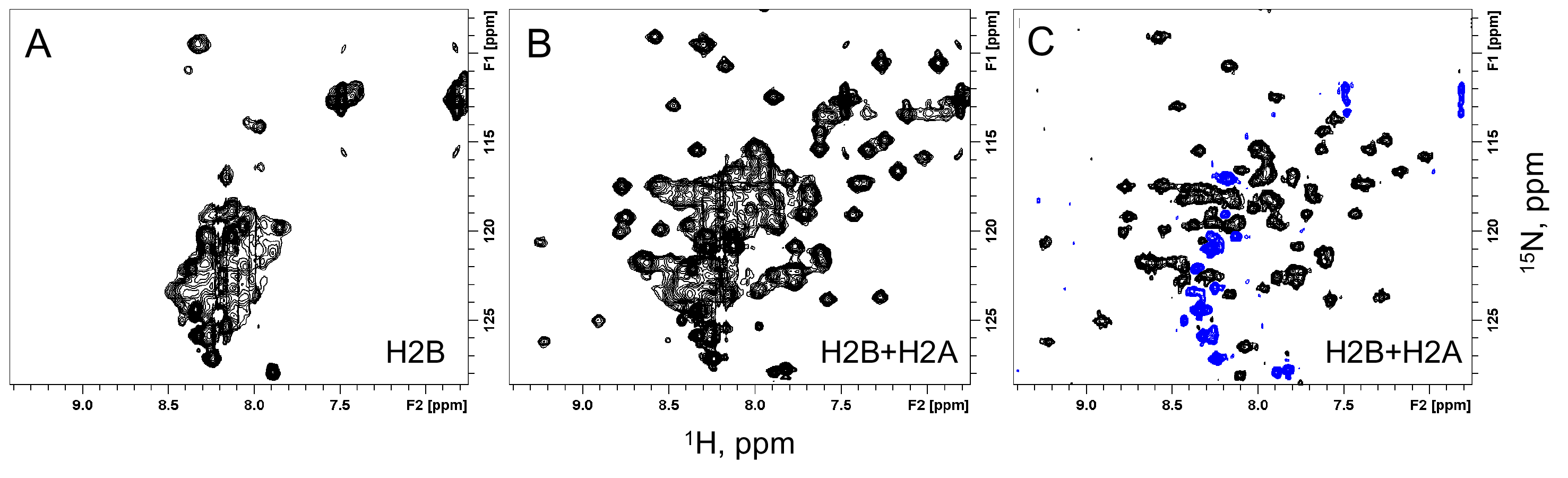

Supplement: S10 Fig — (A) Comparable stability of the inverted non-native conformation is consistently found to that of native structure in simulation replica 2. (B) The initial and final conformation (blue vs. orange) of the inverted structure display minor structural changes before and after 800-ns MD simulations. (TIF) [file pcbi.1011721.s012.tif]

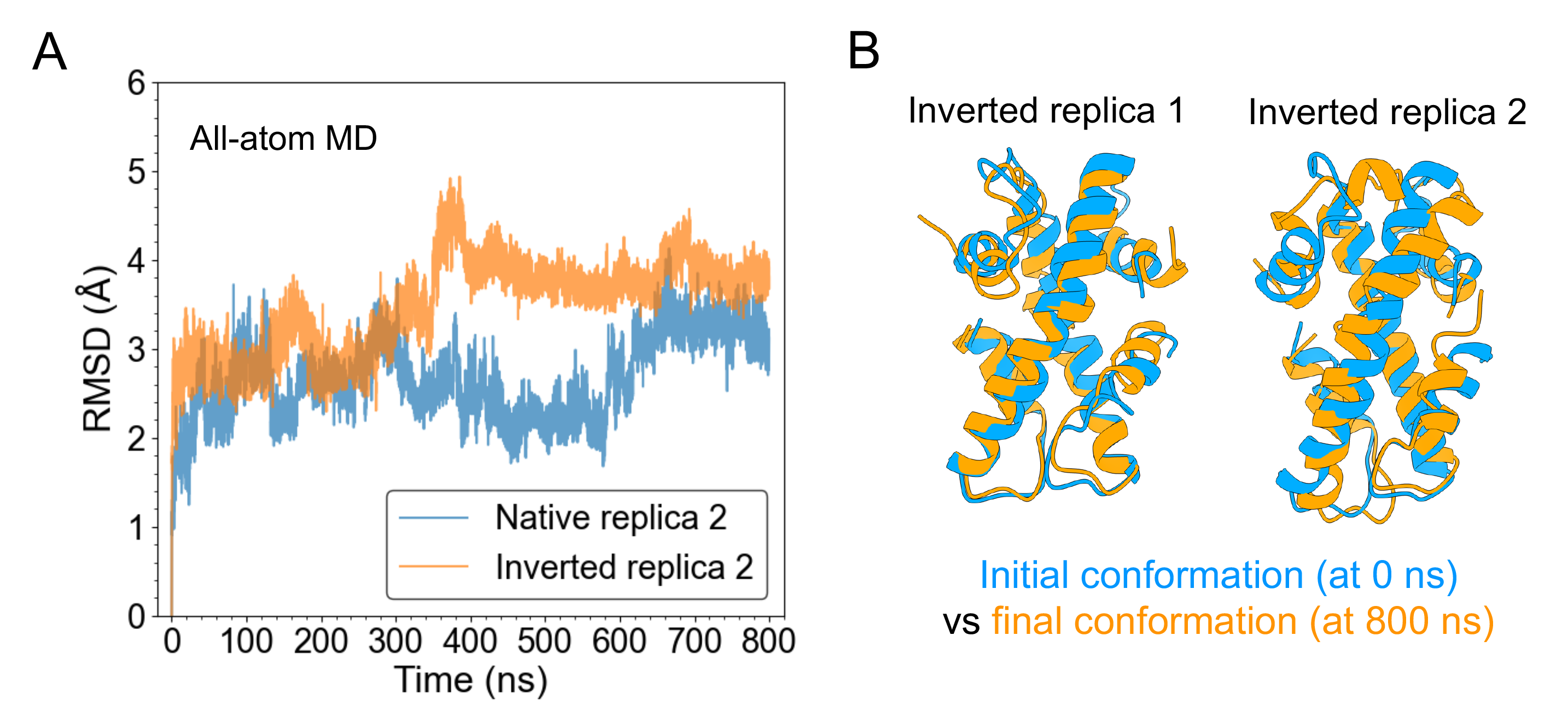

Supplement: S11 Fig — The RMSF analysis of all-atom simulations exhibit similar atomic flexibilities of AWSEM- and AlphaFold2-predicted homo-complex structures in different simulation replicas. The RMSF of two chains are plotted separately where their helix regions are schemed by cartoon in the middle. (TIF) [file pcbi.1011721.s013.tif]

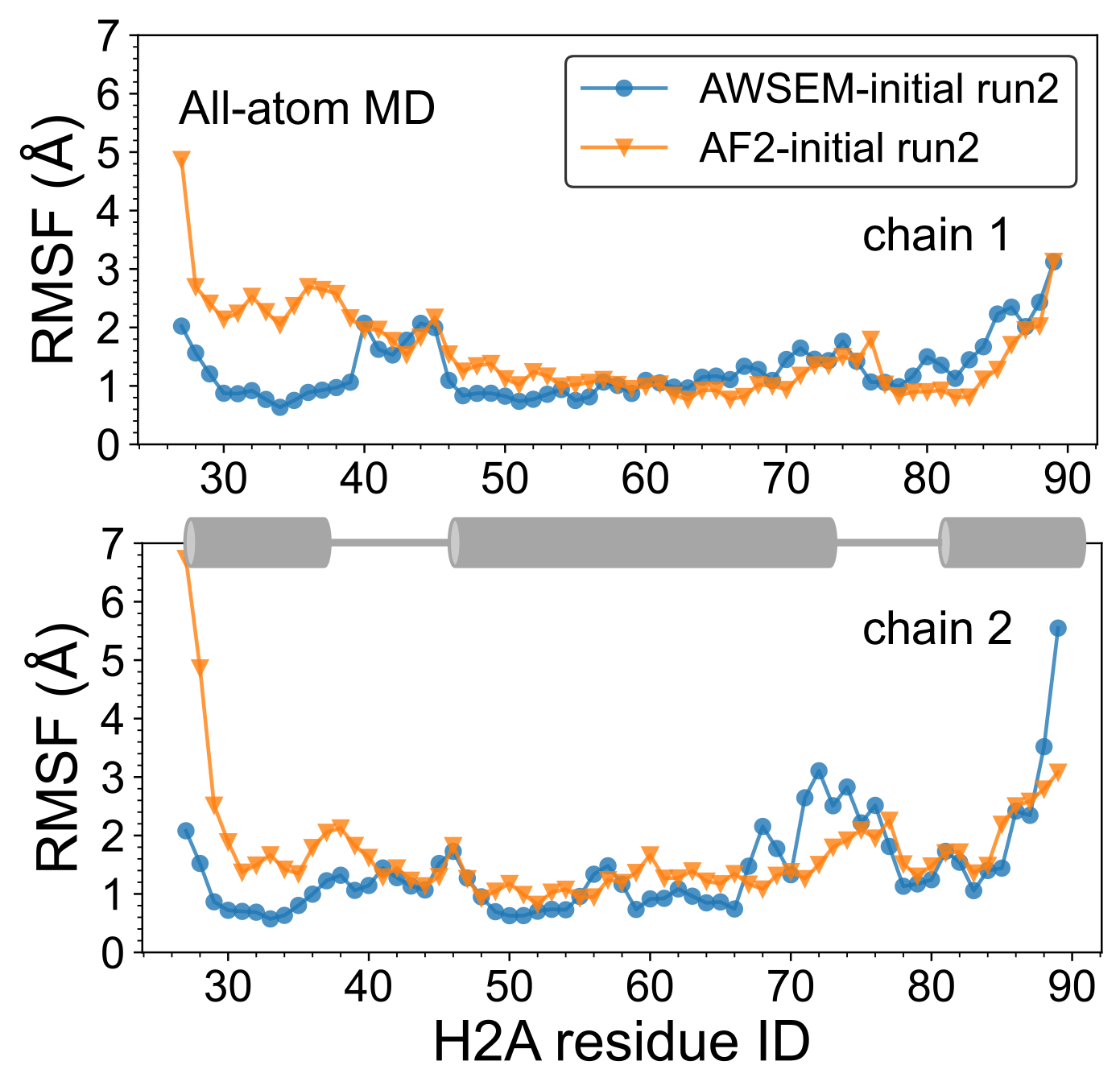

Supplement: S12 Fig — Q value of the histone-fold core region was calculated for the simulated annealing runs of four systems: H2AH2A truncated and full-sequence (colored in blue and orange), H2AH2B truncated and full sequence (green and read). The run that forms the highest Qdimer was chosen for each system. It shows that H2AH2B full sequence forms the most native-like dimer, more than H2AH2A truncated, H2AH2B truncated, and lastly the H2AH2A full sequence. Note that the formed Qdimer of H2AH2B is smaller than that in the dimer folding study as in Fig 2. This is due to the different fragment memory setup. Here, we want to “predict” homodimer structure of H2AH2A so the fragment memory excludes any homolog sequence with 95% identical to the target. To keep consistency, the same setup was used for the control group H2AH2B, which is different from the fragment memory setup in their dimer folding mechanism study. (TIF) [file pcbi.1011721.s014.tif]

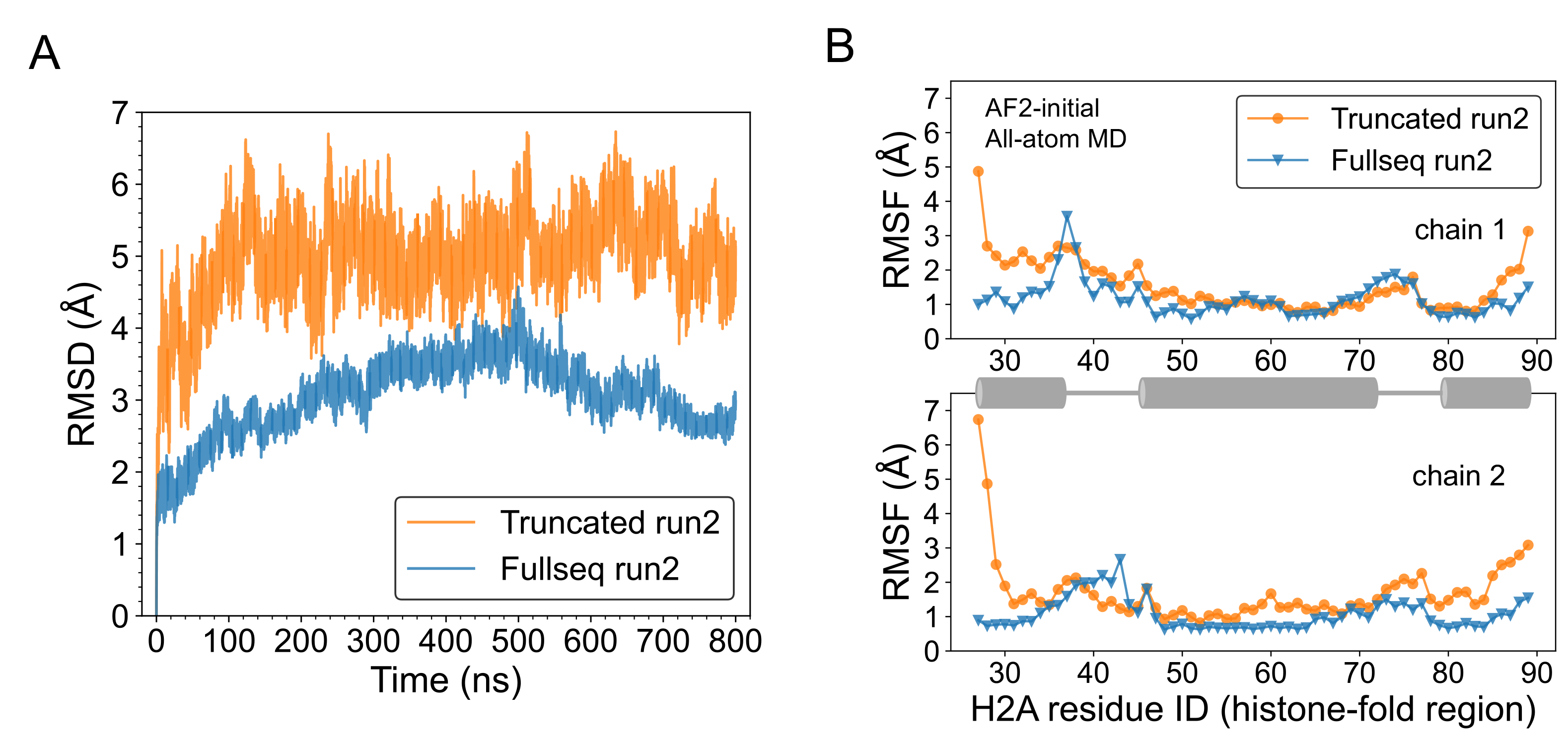

Supplement: S13 Fig — (A) The histone-fold region in full-sequence homodimers has less RMSD (blue) than that of histone-fold only structures (orange) in different simulation runs. (B) The RMSF analyses in different runs demonstrate that the two ending sessions are particularly flexible in truncated homodimers. (TIF) [file pcbi.1011721.s015.tif]

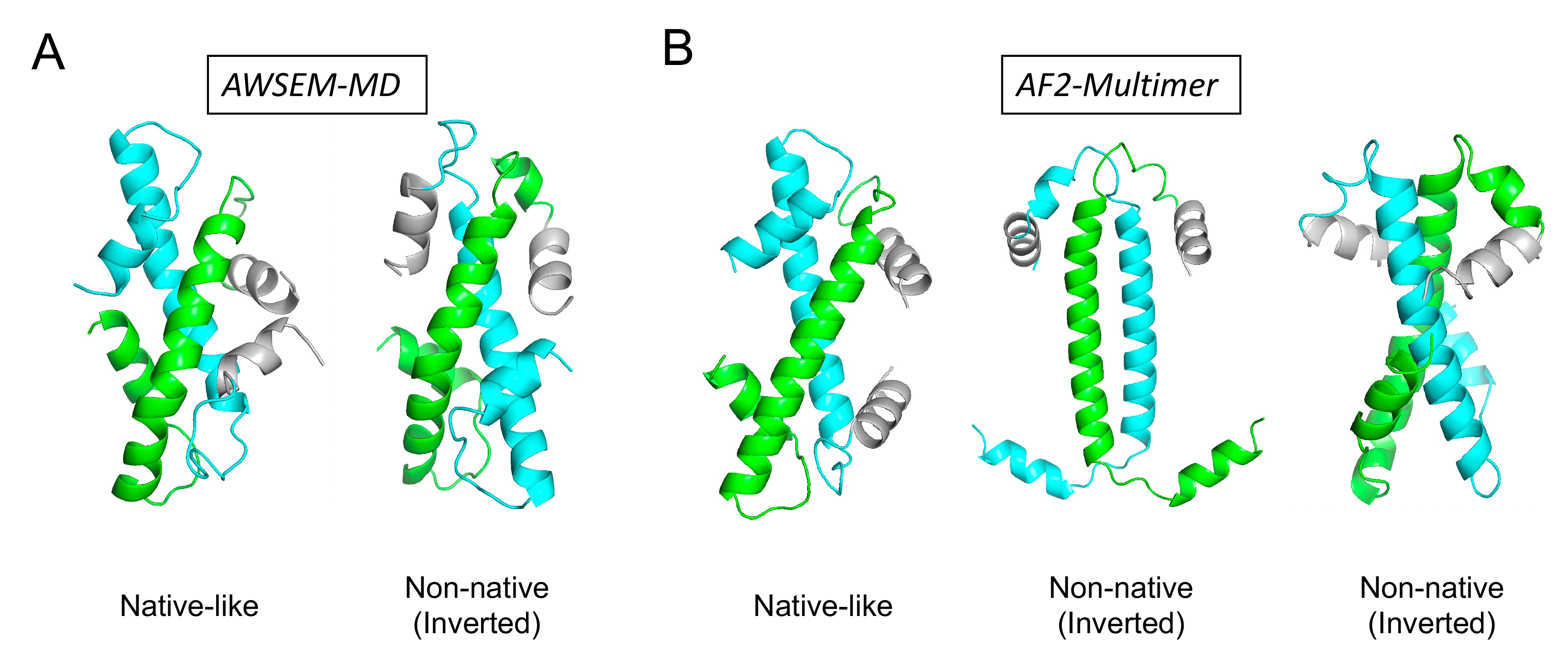

Supplement: S14 Fig — Predicted structures of H2A/H2A by AWSEM (A) and AlphaFold2 (B) are shown, respectively. The two chains are colored in green and cyan while their α1 helixes in grey to help illustrate their native or non-native arrangements. (TIF) [file pcbi.1011721.s016.tif]

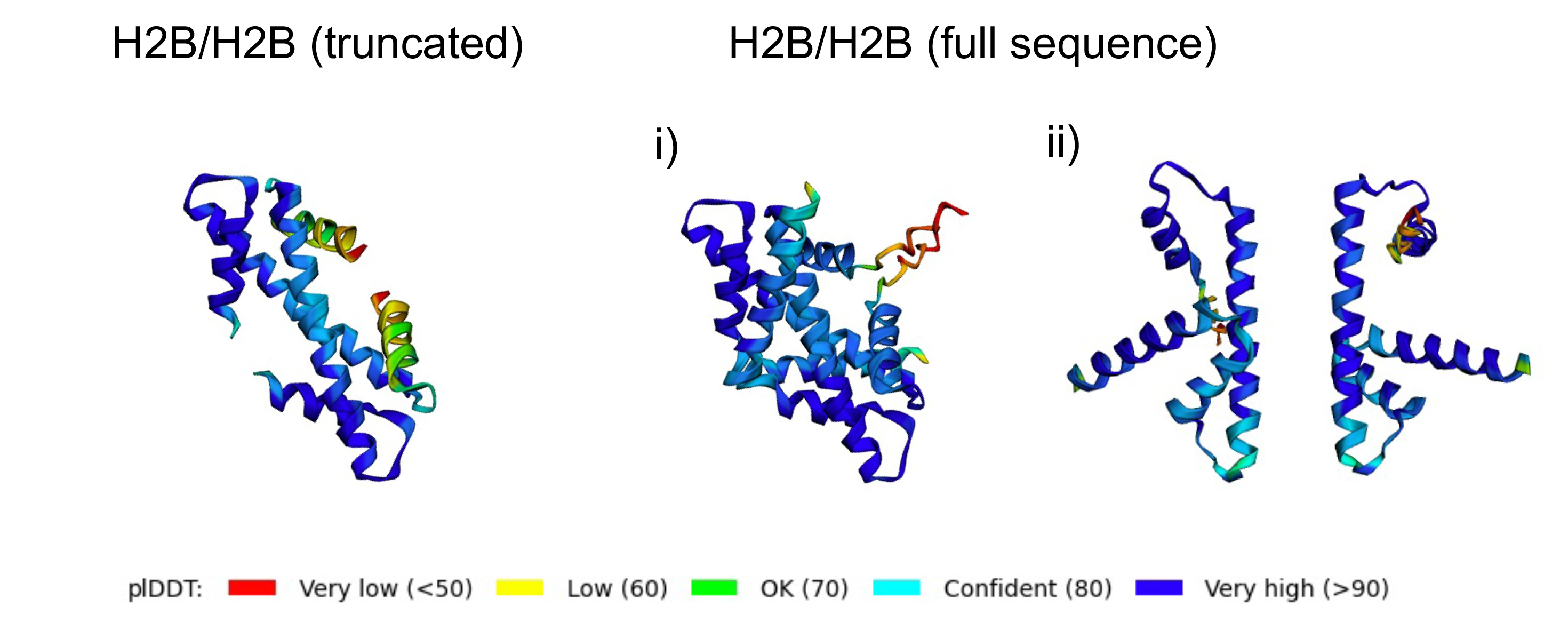

Supplement: S15 Fig — The two chains are colored by the same scheme which is the plDDT confidence score provided by AF2. Both truncated and full-sequence H2B/H2B are predicted to have a native-like handshake structure while juxtaposing monomers are also found (full-sequence structure ii) which potentially indicates two non-interacting monomers. (TIF) [file pcbi.1011721.s017.tif]
