# Supplementary material for: The role of cryptic ancestral symmetry in histone folding mechanisms across Eukarya and Archaea
Source: PLoS Comput Biol. 2024 Jan 5;20(1):e1011721. doi: 10.1371/journal.pcbi.1011721 (PMC10796010; doi:10.1371/journal.pcbi.1011721)
Supplement: Attachment — Submitted filename: Responses_PlosCB_final.docx [file pcbi.1011721.s018.docx]

Reviewer #1: In the present submission “The Role of Criptic Ancestral Symmetry In Histone Folding Mechanisms Across Eukarya and Archaea” H . Zhao et al. investigate homo- and heterodimeric folding for 11 different histone proteins. They apply a range of methods (coarse-grained and all-atom MD simulations, NMR and CD experiments) with a focus on theoretical/ simulation studies. They observe a structural competition of non-native symmetrically related dimeric folds (“native” vs. “inverted”) for tail-less histones.

Summed up, histone folding mechanisms are a topic of high biological interest and there is some discussion regarding their evolutionary changes. The present submission itself is well-written with a clear line of thought and investigate histone proteins in high detail. The technical aspects are well described. There are only few parts which need some minor work and should be addressed before publication (see below). After minor revision, the submission is suitable for publication.
We thank the reviewer for their encouraging words and helpful comments!
Major Issues:
-
Minor issues:
-p. 5, methods and p.7 results: Any stochastic search for a global minimum can instead result in only identifying local approximations of the global minimum or even more dissimilar low energy structures. The author should include a short discussion how reliably using simulated annealing is when being applied on the AWSEM forcefield.

We agree with the reviewer about the general challenge in finding the global minimum. Simulated annealing is a powerful stochastic optimization algorithm used to search for the global minimum using a cost function such as the conformational energy. AWSEM, the associative memory, water mediated, structure and energy model, is a transferrable coarse-grained protein model deeply based on the funneled energy landscape theory of protein folding. In previous works, simulated annealing has been extensively used and tested for its satisfactory efficiency with AWSEM force field in predicting protein structure and protein-protein interface.

In the revision, we added the following text on page 3: “*AWSEM, the associative memory, water mediated, structure and energy model, is a transferrable coarse-grained protein model deeply based on the funneled free energy landscape of protein folding. With the simulated annealing procedure, AWSEM has undergone extensive tests for its satisfactory capability in predicting protein structure and protein-protein interface {\cite{davtyan2012awsem,zheng2012predictive}”.*

-p. 6 “complete all-atom results are in S3.2”. should be replaced by something akin to “A detailed setup of the MD simulations is listed in in S3.2”.

We thank the reviewer for the suggestion. In page 6, paragraph 2, we revised the last sentence to “*A detailed setup of the all-atom MD simulations and its complete results are in S3.2*”. Similarly, we have rephrased the coarse-grained modeling part, in the same page, to “*A detailed setup of the AWSEM model and its complete simulation results are in S3.1*”.

-p. 10 “FE Landscape” might be replaced by “Free Energy Landscape” as it is a heading.

The mentioned abbreviation in the heading has been changed to “Free Energy Landscape”.

-p.13 Fig. 4a, the two dimeric chains could be colored differently for clarity.

The two dimer chains in Fig. 4a have been colored differently.

-p.14 Last paragraph “in an unrelated protein, the Rop dimer, was…” Symmetrically related folds have been a recurring topic in the protein folding literature also for other cases than the Rop-dimer (e.g. mirror folds, J. Noel et al., J. Phys. Chem. B 2012, 116, 23, 6880–6888) and might be discussed in more broad terms.

We greatly appreciate the reviewer’s valuable opinion about expanding our discussion on protein folding mechanisms, following which we have added an entire paragraph in page 19: the below discussion content:

*“Our study shows that the eukaryotic histone dimers not only have the same structural motif as the archaeal histones, but also share the same folding-upon-binding mechanism, indicating that histones form obligatory dimers{\cite{levy2004protein}}. The presence of their binding partner may largely stabilize the mobility of folded secondary structures. Our further finding of energetically favorable native and inverted dimers points to the hidden ancestral symmetry of histone sequences. Not only are the conformation and sequence symmetry revealed here self-consistent, but more broadly, they are consistent with the symmetry-related folds, a recurring topic in protein folding studies {\cite{wolynes1996symmetry,andre2007prediction,noel2012mirror,levy2005symmetry,kachlishvili2014accounting}}. Onuchic and colleagues studied the mirror image folds of three-helix bundle proteins {\cite{noel2012mirror}}. Levy and Wolynes suggested a double-funneled energy landscape model to unravel the folding symmetry of a dimer protein{\cite{levy2005symmetry}}. Baker and colleagues developed a computational protocol to predict the structure of symmetrical protein assemblies and further a method to design protein homodimer based on the cyclic two-fold symmetry {\cite{andre2007prediction,hicks2022novo}}. In this context, the consistent symmetry between sequence and structure uncovered in our study may aid in unraveling the complexities of histone-like structures and functions, offering insights for further applications in structural biology and protein design.”*

Reviewer #2: This is an interesting study trying to shed some light on the evolution of structure and folding mechanisms of H2A/H2B dimer. In this respect, the title and abstract of the paper could be misleading and have to be renamed specific to H2A/H2B, other histone types have not been investigated. Overall paper is well written and has wonderful illustrations. The authors have observed the folding upon binding of H2A-H2B dimer in silico and experiments, this section is very solid. The finding of the inverted intermediate is also fascinating. I have several comments and suggestions which could approve the paper and make it clearer.

We thank the reviewer for their supportive comments and appreciation of the work! In this work, we studied the folding mechanism of histones for 8 types of histone dimers (summarized in Fig 1C) with consistent findings including the inverted conformation. Because of the expensive computation of all-atom simulations, we only took H2A/H2B as an example to study the homodimer formation and their tail effect. To make this point clear, we added result of archaeal histones from SI to Fig 3C, and emphasized the result availability of other histones and histone-like transcription factors in page 11.

My main comment relates to the last result on histone tails’ contributions to the stabilization of the H2A/H2B heterodimer and destabilization of a homodimer. This finding and its discussion are rather vague. There could be several factors at play to explain this result:
- 1) tails might not “tip the balance” if the homodimer is rearranged into “inverted” head-head conformations.
- 2) As H2B tails contain negative charges unlike H2A tails, there is a possibility that tail-tail H2A-H2B interactions may stabilize the heterodimer.
- 3) The authors applied AWSEM which uses the globular native protein fragments for biasing – were these fragments derived from heterodimers and histone octamers rather than from monomers, could it explain their results?
- 4) Can the authors include plots for dimers with tails: Q vs annealing temperature?
- 5) How relevant is this result to histones that function in the nucleosomal context? H2A/H2B histone tails in nucleosomes have different interaction and stabilization scope.

We greatly thank the reviewer for careful considerations of factors that may explain our histone tail result. Below we respond to these factors one by one:

tails might not “tip the balance” if the homodimer is rearranged into “inverted” head-head conformations.

1). We agree with the reviewer that if doing all-atom simulations with the initial configuration of a homodimer in an “inverted” conformation, it is possible that we may not observe tails to “tip the balance”. Observing protein refolding is usually estimated on the timescale of milliseconds, which is very challenging to reach for atomic MD simulations in practice, especially for dimer proteins here. Thus, our conclusion for the “tipping” role of tails is mainly based on the coarse-grained simulations of histone homodimers with the AWSEM protein model (Fig. 5). In the simulated annealing runs with AWSEM, all proteins start with completed unfolded states, like random coils, wherein the geometry and orientation are not clearly defined.

As H2B tails contain negative charges unlike H2A tails, there is a possibility that tail-tail H2A-H2B interactions may stabilize the heterodimer.

2). We thank the reviewer for this point. We provided the sequence of histone tail in the SI S3.2, Fig S1.B. The C-terminal H2B tail does contain several negative charges, which may stabilize the heterodimer formation as the N-terminal H2B tail and both H2A tails are all positively charged. We have added this explanation in the discussion on page 20.

*“Interestingly, unlike most histone tails, the C-terminal tail of H2B contains several negative charges which may favor formation of a heterodimer versus a homodimer.”*

The authors applied AWSEM which uses the globular native protein fragments for biasing – were these fragments derived from heterodimers and histone octamers rather than from monomers, could it explain their results?

3). We greatly appreciate the reviewer for understanding and asking this technical detail. Very different from GO potential, all the fragments used in the AWSEM potential are local fragments range from 3 to 9 amino acid long. There is no biasing term for any intermolecular interactions. Regarding the concern on the homodimer prediction, the fragment memory used there are not necessarily from histones. Mostly they are fragments of homolog sequences with sequence identify below 95% to the target. We now emphasized the technical detail in two places, in Methods (page 5-6), and the result session of homodimer (page 18). Respectively, the edits are:

*“In this work, the fragment library used in the last term is composed of local fragments (3-9 residues long) either from the structure of histone monomers for the dimer folding study, or called "homologs-allowed", from the structural fragments of their homologous sequences with less than 95% sequence identity to the target. The latter was used to explore possible monomer and homodimer structures. The control group of heterodimer in the homodimer study used the same setup.”*

*“Twenty independent simulated annealing runs were performed in AWSEM using the fragment memory of their homologs-allowed sequences (<95% sequence identity to target; details see Methods) for each system.”*

Can the authors include plots for dimers with tails: Q vs annealing temperature?

4). We have added a new plot (Figure S9) of Q vs annealing temperature for the dimers with tails in the SI and added the below description and discussion.

*“Q value of the histone-fold core region was calculated for the simulated annealing runs of four systems: H2AH2A truncated and full-sequence (colored in blue and orange), H2AH2B truncated and full sequence (green and read). The run that forms the highest Q_dimer was chosen for each system. It shows that H2AH2B full sequence forms the most native-like dimer, more than H2AH2A truncated, H2AH2B truncated, and lastly the H2AH2A full sequence. Note that the formed Q_dimer of H2AH2B is smaller than that in the dimer folding study as in Figure 2. This is due to the different fragment memory setup. Here, we want to "predict" homodimer structure of H2AH2A so the fragment memory excludes any homolog sequence with 95% identical to the target. To keep consistency, the same setup was used for the control group H2AH2B, which is different from the fragment memory setup in their dimer folding mechanism study.”*

How relevant is this result to histones that function in the nucleosomal context? H2A/H2B histone tails in nucleosomes have different interaction and stabilization scope.

5). We agree with the reviewer that H2A/H2B histone tails in nucleosomes have different interaction and stabilization scope. Our current study focuses on the folding mechanics of histone dimers themselves. We added a short discussion regarding this concern in page 20.

*“Histone tails have undergone extensive investigation for their impact on nucleosome stability and binding with other chromosome regulator proteins. Our study here delves into the folding dynamics of histones before they form nucleosomes. It is expected that their dynamics may differ once in a nucleosomal context.”*

“A scaling analysis of the radius of gyration (Rg) as a function of the protein size suggests
that histone dimer acts as the minimum folded state, rather than its monomer” – the authors probably mean that histones form obligatory dimers?

Yes, we meant that histones form obligatory dimers. We rephrased the related context to the below:

*“A scaling analysis of the radius of gyration (Rg) as a function of the protein size shows that histone dimer fits almost perfectly to the empirical scaling relation of monomeric proteins, suggesting that histones may form obligatory dimers.”*

We also mentioned the obligatory dimers in the discussion:

*“Our study shows that histone dimers not only have the same structural motif across eukarya and archaea, but also share the same folding-upon-binding mechanism which indicates that histones form obligatory dimers.”*

“Our simulation indicates that histone monomers are highly disordered at the tertiary structure level” – they are mobile, but not disordered, at least not intrinsically disordered.

We thank the reviewer for notifying the accuracy of expressions. We have changed the related context: “*Our simulation indicates that histone monomers are highly mobile at the tertiary structure level.”*

Figure 4 and Figure S2. Please say which program was used to align these sequences or structures.

We have used MUSCLE v3.8.31. The related methods details were originally in the supplemental information. We now moved the related contents to the Methods of the main context:

*“All used protein sequences in this study are downloaded from UniProtKB database {\cite{uniprot2019uniprot}} and aligned using MUSCLE v3.8.31 {\cite{edgar2004muscle}}. The generated multiple sequence alignments are visualized by an online tool, Multiple Align Show, $https://www.bioinformatics.org/sms/multi\_align.html$. Structure and simulation conformation figures used in this work are generated by PyMOL Molecular Graphics System Version 2.3.2, Schr\"odinger LLC.”*

Were the distance constraint applied for dimers and not for monomers? If yes, could it potentially bias the results on folding mechanisms?

We used a weak distance constraint in the dimer study. It is in a harmonic potential form with the spring constant as 0.02 (kcal/mol)/Å^2^ and was applied to the center of mass of each protein. This potential mimics a finite protein concentration. Because AWSEM simulations are represented within free boundary conditions (i.e. non-periodic), one expects from statistical mechanics that without a constraint between centers of masses two proteins will always drift way from each other. Considering that it is a weak constraint and applied to the entire protein, we do not expect it may bias the result on the folding mechanism. For clarification, we moved the related description from the SI to the main context, now in Methods session, page 6:

*“To maintain a reasonable contact region, a weak distance constraint in the format of harmonic potential was applied between the centers of mass of two simulated monomers (the spring constant $k$ = 0.02 kcal/mol/\AA$^2$).”*

How “completely unfolded state” was generated?

The system was initialized at constant 1000K for running 30,000 timesteps to generate the completely unfolded state. We added this technical step in the Methods session, page 6.

*“The initial conformations of the unfolded state were prepared at 1000K.”*

Folding of dimers is shown as a function of the annealing temperature in AWSEM-MD simulations – it would be beneficial (although probably time consuming so I do not insist on this) if the authors can apply their method to a dimer with monomers known to be structured in unbound state – for control.

We thank the reviewer for this suggestion. However, because we are not aware of any histone monomer protein that can fold on its own, such additional simulations would indeed be time consuming without having direct connection to experiment.

Folding upon binding mechanisms, for some reason, are not even mentioned in Discussion.

Indeed, we totally agree and thank the reviewer for bringing up this point. We now have added an entire paragraph to discuss the folding upon binding mechanism which may be interesting to the general biophysics field and inspiring to protein design.

*“Our study shows that the eukaryotic histone dimers not only have the same structural motif as the archaeal histones, but also share the same folding-upon-binding mechanism, indicating that histones form obligatory dimers{\cite{levy2004protein}}. The presence of their binding partner may largely stabilize the mobility of folded secondary structures. Our further finding of energetically favorable native and inverted dimers points to the hidden ancestral symmetry of histone sequences. Not only are the conformation and sequence symmetry revealed here self-consistent, but more broadly, they are consistent with the symmetry-related folds, a recurring topic in protein folding studies {\cite{wolynes1996symmetry,andre2007prediction,noel2012mirror,levy2005symmetry,kachlishvili2014accounting}}. Onuchic and colleagues studied the mirror image folds of three-helix bundle proteins {\cite{noel2012mirror}}. Levy and Wolynes suggested a double-funneled energy landscape model to unravel the folding symmetry of a dimer protein{\cite{levy2005symmetry}}. Baker and colleagues developed a computational protocol to predict the structure of symmetrical protein assemblies and further a method to design protein homodimer based on the cyclic two-fold symmetry {\cite{andre2007prediction,hicks2022novo}}. In this context, the consistent symmetry between sequence and structure uncovered in our study may aid in unraveling the complexities of histone-like structures and functions, offering insights for further applications in structural biology and protein design.”*

Lastly, regarding the concern of data repository:

We originally uploaded our data and code in the zenodo but did not mention it in the context. Now we added the below description in page 23.

*“Data and Code Available:*

*The molecular dynamics simulation data, used tools, and scripts for this study are uploaded on zenodo server, https://zenodo.org/records/8226152. The simulation data includes the force field and simulation setup for both AWSEM-MD and atomistic MD in OpenMM, the data analyses that are presented in the paper, and the representative conformations from each simulation. Due to the large file size, the original trajectory files for the many simulations done here are available upon separate request.”*

Reviewer #3: The manuscript presents and interesting computational stud on the folding and binding mechanism of formation of histone proteins. The study is comprehensive in terms of the systems that are studied and the methods. It also combines some experimental results.

We thank the reviewer for noting the comprehensive nature of this work!

Comments
1. It seems that the presented study reports on two major results: the inverted symmetry due to the “quasi symmetric sequences” and its effect on the inverted structure and the effect of the tails. While the former is discussed in length, *the latter is barely discussed. This asymmetry should be somehow addressed. There are no sequences of the tails, their length. No conformations of the tails and how they may interfere with folding. Also the references on the effect of disordered tails on folding or binding is limited*.

We thank the reviewer for these suggestions. In this work, we mainly focused on the folding mechanism of histone-fold structures. We reported an “unexpected” inverted conformation which leads to unravelling of the head-to-tail symmetry of histone sequences. We then studied the role of histone tails in their folding of the homodimer H2A/H2A. Now we added more data analysis including the role of histone tail in the formation of heterodimer H2A/H2B (Figure 6A, 6B).

*“Twenty independent simulated annealing runs were performed in AWSEM using the fragment memory of their homologs-allowed sequences (<95% sequence identity to target; details see Methods) for each system. It is evident that in most annealing runs, histones without tails form higher Qdimer compared to those with tails (Figure 6A), suggesting a significant inhibitory effect of histone tails in their dimer formation, whether in histone heterodimer or homodimer. In the presence of tails, the formation rate of histone heterodimers is notably higher than that of homodimers, corroborating our hypothesis that histone tails play a pivotal role in tipping the balance in favor of heterodimer formation.”*

*“We then performed all-atom MD simulations by taking the predictions of AF-Multimer for H2A/H2A homodimers, both with and without histone tails, as initial conformations and compared them with simulations of H2A/H2B in similar cases (full-sequence vs. truncated). The RMSD analyses confirmed that the predicted H2A/H2A structures, with truncated tails or not, are less stable than the corresponding H2A/H2B structures. Furthermore, interestingly, the full-sequence H2A/H2A and H2A/H2B have lower RMSD (histone-fold part), on average, than the truncated H2A/H2A or H2A/H2B (Figure 6B). RMSF analysis (Figure 6C) shows that the flexibility difference between the truncated and full-sequence systems mainly appears at the terminal residues of the histone-fold core and the loop regions. Together, our simulations suggest that once the full-sequence H2A/H2A or H2A/H2B has formed a histone-fold core, the disordered tails may potentially help stabilize the loop regions.”*

Following the reviewer’s suggestion, we added the sequence and length of histone tail in the SI session S2, Figure S1B. Lastly, in the main context, we added an entire paragraph to discuss histone tails and included more citations on histone tails. We apologize for missing related works in the first place!

*“From a biology perspective, an interesting consequence of eukaryotic histones having forked from the archaeal tree is that eukaryotic histones have disordered tails and do not form homodimers. Our \textit{in silico} results suggest that eukaryotic histones may form homodimers as well, with histone tails potentially destabilizing this formation. Positively charged histone tails are typically involved in binding to DNAs or various chromatin-modifying enzymes and transcription factors. Interestingly, unlike most histone tails, the C-terminal tail of H2B contains several negative charges which may favor formation of a heterodimer versus a homodimer. However, a previous experimental work pointed that electrostatic repulsion in the N-terminal tails decreases the stability of the H2A-H2B dimer \cite{placek2002n}. Future investigations could explore the influence of specific terminal tails of certain histones on the formation of homodimers or heterodimers. Besides the sequence and varying lengths of histone tails, their post-translational modifications may also affect the overall dynamics of histones and nucleosome{\cite{bannister2011regulation,potoyan2012regulation,winogradoff2015acetylation}}. Histone tails have undergone extensive investigation for their impact on nucleosome stability and binding with other chromosome regulator proteins {\cite{iwasaki2013contribution,li2016distinct,kameda2019histone,morrison2018conformation,biswas2011role}}. Our study here delves into the folding dynamics of histones before they form nucleosomes. It is expected that their dynamics may differ once in a nucleosomal context.”*

2. The impression is that most of the results are stored in the SI. Particularly the results of some of the systems. The authors may consider to move some of the results to the main text.

We totally agree with the reviewer and appreciate the review’s careful reading of our SI. As a work of many years’ efforts, we have done a significant number of simulations and analyses. Due to the large volume, we find it extremely challenging to place all the results in the main text. Despite this, we moved part of Figure S6 to Figure 2, Figure S7 to Figure 3B-C. We also added structure representations in Figure 6. Meanwhile, we added several places to remind readers that there are significantly more results are in the SI like:

In page 11, *“Not only in eukaryotic histones (Figure 3B), this inverted conformation was also found in other simulated histone-fold sys- tems, including archaeal histones (Figure 3C) and transcription factors dTAFII42/dTAFII62 dimer and NF-YB/NF-YC dimer (Figure S7; also see the SI movie for dynamics details).”*

in page 22, *“Extensive data analyses and method details, including the following, are provided in the Supporting Information.”*

In page 23-25, we added detailed contents in the SI.

3. Figure 4 is important but its caption is not clear enough. Particularly the relationship between (A) and (C)ii. Also shouldn’t some of the termini be labeled as C and N (without ‘)?
Also, it might be useful to show the location of the tails on these schemes?

We are grateful to the reviewer for this comment. We have changed the labels of termini to C and N. Figure 4 shows a set of cartoon schemes of the histone-fold structures regardless of species, wherein the existence and length of histone tails vary. We added more details about the histone evolution hypotheses in the below context and also edited the figure caption to make it clearer. Changes are marked in red.

*“The finding of head-tail sequence symmetry and inverted histone dimer well support the previously proposed histone evolution hypotheses. The latter posits that histones may have arisen from one common ancestral peptide, the helix-strand-helix (HSH) structural motif (Figure 4C i). Through their duplication, differentiation and fusion in the context of RNA-dependent replication and catalysis (Figure 4C ii), two HSH patterns may form one histone monomer and domain swapping may have triggered the subsequent dimerization of two histone monomers (Figure 4C iii). Thus, it is possible that the conservation of hydrophobic positions found in histone sequences, no matter in a normal or reversed order, originated from the same ancestral peptide. Meanwhile, the conserved hydrophobicity in the four segments of two histones explains the rationale of two alternative ways of the crossing forming the hand-shake motif for the characteristic of the histone fold.”*

Figure 4 caption: *“Sequence symmetry of hydrophobic residues explains the predictability of inverted histone-fold structure. (A) Hydrophobic interactions (orange) dominate the formation of binding interface of H2A/H2B (black/silver). (B) Protein sequence alignments of histones H2A, H2B and their reversed sequences highlight the symmetrical distribution of hydrophobic residues. Hydrophobic residues are particularly marked. (C) Cartoon schemes illustrate previously proposed histone evolution hypotheses: i) histones may originate from one single helix-strand-helix (HSH) structural motif; ii) two HSH patterns form one monomer through duplication, differentiation and fusion; iii) domain swapping between two monomers (colored in green and blue) forms a histone-fold structure. (D) The inverted arrangement based on hydrophobic interactions could be an alternative formation of histone-fold structure.”*

4. Conclusions: One sentence says: “ without tails may form a low-energy non-native dimeric complex….” And another sentence “without tails, eukaryotic histones form potentially stable homodimers…”. One may read these as contradictory. A revision/clarification will be useful.

We thank the reviewer for suggesting a need for clarification. We have changed the related text to the one below:

*“Moreover, histone-fold structures may form a low-energy non-native dimeric complex, in which on the tertiary structure level, an inverted arrangement is energetically nearly competitive with the native conformation. … Without tails, eukaryotic histones form potentially stable homodimers in both AWSEM and AlphaFold-Multimer predictions, while with tails AWSEM simulations indicate an impeded formation of histone homodimers. Overall, our research illustrates common folding mechanisms of histone proteins, shedding light on their ancestral sequence and structural symmetries, and various new asymmetries engendered by evolution.”*
